# Exploratory behaviour in NO-dependent cyclase mutants of *Drosophila *shows defects in coincident neuronal signalling

**DOI:** 10.1186/1471-2202-8-65

**Published:** 2007-08-06

**Authors:** Sylvette Tinette, Lixing Zhang, Amélie Garnier, Gilbert Engler, Sophie Tares, Alain Robichon

**Affiliations:** 1 Université de Bourgogne 15 rue Hughes Picardet Dijon 21000 France CESG/CNRS France; 2 Université de Nice Sophia Antipolis, 400 routes des Chappes 06903 Sophia Antipolis INRA/CNRS France

## Abstract

**Background:**

*Drosophila *flies explore the environment very efficiently in order to colonize it. They explore collectively, not individually, so that when a few land on a food spot, they attract the others by signs. This behaviour leads to aggregation of individuals and optimizes the screening of mates and egg-laying on the most favourable food spots.

**Results:**

Flies perform cycles of exploration/aggregation depending on the resources of the environment. This behavioural ecology constitutes an excellent model for analyzing simultaneous processing of neurosensory information. We reasoned that the decision of flies to land somewhere in order to achieve aggregation is based on simultaneous integration of signals (visual, olfactory, acoustic) during their flight. On the basis of what flies do in nature, we designed laboratory tests to analyze the phenomenon of neuronal coincidence. We screened many mutants of genes involved in neuronal metabolism and the synaptic machinery.

**Conclusion:**

Mutants of NO-dependent cyclase show a specifically-marked behaviour phenotype, but on the other hand they are associated with moderate biochemical defects. We show that these mutants present errors in integrative and/or coincident processing of signals, which are not reducible to the functions of the peripheral sensory cells.

## Background

We have shown that exploration in *Drosophila *is a powerful behavioural tool for analyzing the genetic basis of the machinery involved in the integration of multiple environmental signs [1]. We therefore designed protocols to test the exploratory skills of the flies [1]. We reasoned that the efficiency of exploration depends on the simultaneous integration of neurosensory signals during flight to guide the aggregation of individuals on a specific spot. When a food spot becomes disadvantageous, the flies reset a new cycle of exploration/aggregation [1]. We previously showed that the first flies to find the appropriate food spot (explorers) signal to the others (followers) [1]. Such collective exploration leads to remarkably efficient aggregation. They aggregate to screen sex partners and to lay eggs on the food spot most favourable for sustaining the development of larvae. This cooperative task suggests that costly individual foraging behaviour has not been selected by evolution because encounters would have been random, unpredictable and therefore unfavourable for mating. We assume in this report that "mistakes" in aggregation result from misguided integration of visual, olfactory and acoustic signals during flight and/or the overall exploratory process. We think that this simultaneous processing of neuronal signals can be analyzed experimentally by examining how flies achieve aggregation. These neuronal features include the detection and processing of a sequential and temporal order of signs generated in an unstable environment, to guide the flies' trajectory during flight and "decision making" to occupy a spot durably after landing. In other words, flies will be attracted by a bouquet of smells emitted by sources dispersed in the environment. Flies tend to land on an already-populated spot to maximize aggregation and to ignore "unoccupied" sources emitting the same bouquet. According to a "trade-off" mechanism, a high density population on a spot will prompt re-colonisation somewhere else by a fraction of the aggregated population. If a food spot is attractive because of the odours emitted but turns out to be distasteful and/or poisonous, the aggregate will probably not form. In this report, we designed tests derived from protocols described in our previous publication in order to analyze this stereotyped task [1]. We show evidence that the capacity of the neuronal network to process coincidental signalling can be thoroughly evaluated in this way. Owing to the difficulties of encapsulating all the sensory functions and their integration in an entire body, we focus on the wing nerve, which conveys two major signals to the brain during flight: velocity- and chemo-detection. We used this neuronal system because the circuitry is accessible and the axons of at least two functionally distinct types of neurons (mechanoreceptors and chemoreceptors) are entangled, generating a unique bundle that projects to the same thoracic nervous structure site [2-5]. The two types of sensory information are therefore simultaneously transported via the same nerve. Although our comprehension of the integrative function of the brain in guiding the "decision" in an exploratory task is still incomplete, we report data suggesting that the collective exploration component is very robust; it is preserved in most of mutants tested (see the list of CNS mutants in Additional file 1) but drastically altered in *sGC *mutants. Moreover, NO (activator of *sGC*) has been reported to function as a combined 'slow-down and search signal' for growth cones of neuronal cells and a potent stop signal for developing neurites, which suggests mistakes in neuronal network and/or misguided neuronal wiring in flies lacking *sGC *(see arguments in discussion section). We reasoned that instable synapses and defective wiring should affect the synthesis/transport of synaptic vesicle components. Due to multiple difficulties to investigate whether synapses are appropriately formed at the right places in our mutants, we chose to examine indirectly the axonal transport of synaptic vesicle components in a very accessible nerve: the anterior wing margin nerve.

## Methods

### Strains used in this work

**Figure 1:**
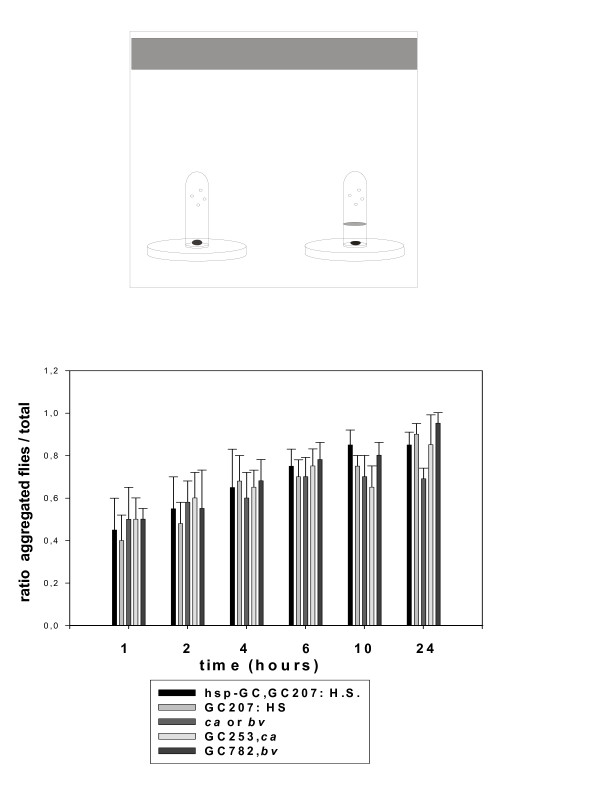
**Comparison between strains to perform aggregation**. The design of the apparatus is drawn in the top panel and the protocol is described in the Methods section. Briefly, we analyzed the kinetics of accumulation of flies in two tubes placed in a chamber. Flies enter through the holes to reach food but in one system there is no access. We measured the time necessary for flies to "correct their mistake" and aggregate in the "reward" tube. Two hundred male and female flies (5 days old) were released into the chamber and the total aggregate (flies in the two systems: the accessible and the inaccessible) *versus *the total in the chamber at each time and for each strain is represented. We observed no statistically significant difference (Student's t test). Values are mean ± SEM (n = 10).

All the strains tested and not retained for further investigations are listed in Additional file 1. The references are indicated for those which are not publicly available. These lines were tested with the protocols described in figure 1. After five experiments showing little effect compared to control (*C-S*) we decided to stop investigating these mutants in the exploratory paradigm. *dnc *and *rut *and the *GFP *constructs P [GawB] elav [C155], P [UAS-syt.eGFP], w* (*Bloomington Center*, strain number 6923) and P [GawB]elav [C155], P [UAS-n-syb.eGFP], w*/FM7 (6923 and 6920) were obtained from the *Bloomington Center*. We focused on the *sGC *mutants due to a strong behavioural phenotype. Three mutants of *sGC *were used: *GC782 *and *GC207 *induced in the *ca *marked chromosome, and *GC253 *induced in the *bv *marked chromosome, have been published [6,7]. The rescue is *hsp-GC *introduced in the *GC207 *background [6,7]. The strains P [GawB] elav [C155], P [UAS-syt.eGFP], w* (*Bloomington Center*, strain number 6923 donator *Kendal Broadie*) and P [GawB]elav [C155], P [UAS-n-syb.eGFP], w*/FM7 (*Bloomington Center*, strain number 6920, donator *Kendal Broadie*) were crossed with *sGC *mutants as indicated below and in legends to figures.

### Behavioural studies

#### Exploration test

**Figure 2:**
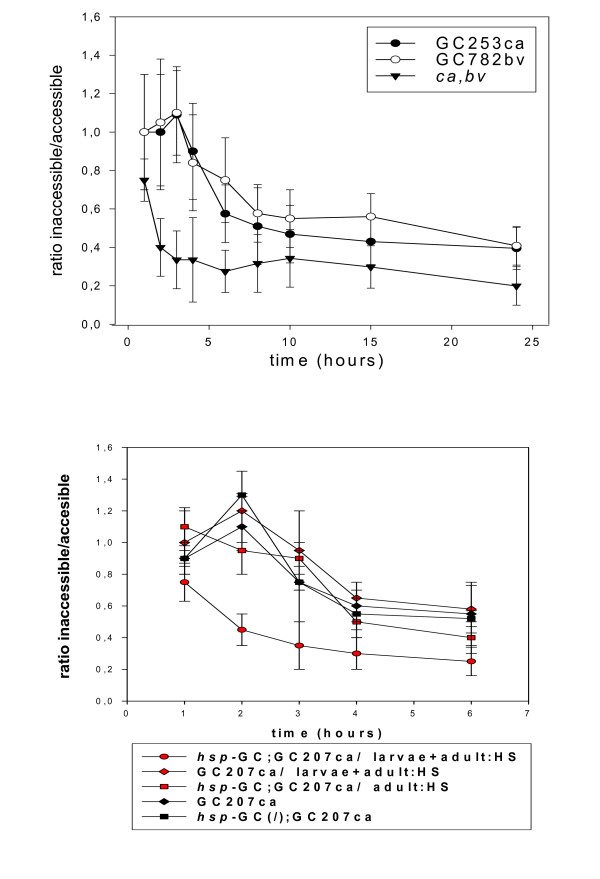
**Performance in exploration is altered in *sGC *mutants**. The protocol is the same than figure 1 and the design of the apparatus used is drawn in the top panel of figure 1. Two hundred male and female flies (5 days old) were released into the chamber and the time-course of the ratio of the number of flies accumulating in the two systems was determined. (*top*) Two mutants, *GC253 ca *and *GC782 bv*, were tested along with controls (*ca *and *bv *showed indistinguishable profiles). Values are mean ± SEM (n = 10) (at 3 h, T value: 4.28, P value: 0.00044, degrees of freedom: 18 for mutants *versus *genetic background). (*bottom*) The mutants and the rescue (*GC207 *and *hsp-sGC; GC207*) were heat-shocked (30 min at 37°C with 3 day old flies), then 5 day old flies were tested. We observed that the rescue did not significantly restore the control phenotype. The rescue was also heat-shocked twice (late third instar larva *plus *adult stage at day 3, same conditions) and this restored the wild type phenotype. Values are mean ± SEM (n = 10) (at 2 h, T value: 9, P value = 0.0001, degrees of freedom: 18 for *hsp-sGC;GC207 versus GC207*, both heat-shocked at third instar larva + adult).

The procedure has been described in a previous report [1]. We used two systems, graphically represented in figures 1 and 2. Briefly, a pierced centrifuge tube is fixed upside down on a Petri dish so that grape juice food inside the assemblage is accessible only by passage through the holes. In the alternative system, such access is prevented by a layer of glass wool. Flies explore and colonize the two systems equally, and over a variable time course they leave the "wrong" system and flock on the "good" one. We described the kinetics of aggregation in a previous article [1], showing a sub-social component of aggregation. Briefly, our data argued in favour of the following behavioural sequence: flies walking on a food spot are moving objects that generate signs (wing motion, proboscis extension, acoustic signs) [8,9]. Other flies land on the spot, attracted by those already aggregated, and avoid having to explore individually.

#### Olfactory/aggregation test

This test uses a chamber and three systems of pierced tubes fixed upside down on a Petri dish as described above. The tubes are painted black to avoid visual cues to guide the flies. Samples of grape juice (50 μl, 100 μl and 300 μl) are placed inside the three systems. The chamber is either saturated with grape juice odorants (hermetic sealing by plastic lid *plus *inaccessible source of grape juice inside as an odour generator) or not (the lid is a synthetic mattress that permits air exchange without the odour generator). After 2, 10 or 24 hours, the flies are counted in each system. This measures the ability of flies to explore in the absence of any odorant gradient or visual perception of conspecifics for guidance.

#### Taste aggregation test

**Figure 5:**
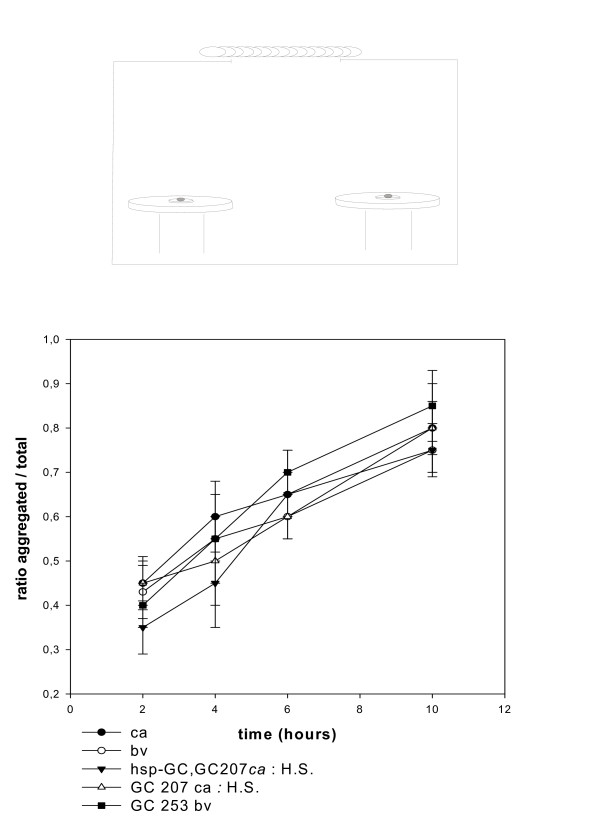
**Aggregation control in the taste/aggregation paradigm **. Flies were placed in the chamber and had to choose between two alternatives to feed (grape juice spot or grape juice/denatonium spot) (see drawing in top panel). The time course of aggregation on the two spots was analyzed with 200 flies. The control for the total aggregated flies (on both spots: grape juice/denatonium *plus *grape juice) *versus *the total in the chamber is represented at each time point. We saw no statistically significant differences between the strains.

This test uses a chamber and two food spots, as represented in figure 5. The two spots are identical grape juice sources, except that one is complemented with denatonium (1 mg/ml) [1]. Denatonium confers bitterness (distasteful for flies as for humans, no perceptible odour) without interfering with the grape juice odorants. We observed a robust behavioural phenotype of aggregation on the source without denatonium for most strains from our shelves (this suggests that flies evaluate the source before they lay eggs on it). We determined the ratio of aggregated flies on the "wrong" spot *versus *the "good" one. We checked bitterness detection with quinine (1 mg/ml), which is less drastic than denatonium because, after tasting, denatonium blocks food uptake for a few minutes.

#### Sub social aggregation test

A complementary test measures the behaviour of a few flies when they face a choice between two food systems that are identical except that one is already aggregated with *w1 *flies (white-eyed flies can easily be discriminated from the red-eyed flies to be tested). The ratio of flies landing on the spot with pre-aggregated *w1 *flies *versus *the virgin spot measures the sub-social component of the strain being tested.

#### Physical lesion of the axonal bundle of the wing margin

The nerve of wings was sectioned with a razor in the proximal part of the wing of *C-S *under C0_2 _anaesthesia. The flies (100) were tested one day after the surgery using a derived aggregation protocol (a chamber *plus *a food spot placed on the bottom).

### Analysis of *syt *and *n-syb *synthesis in *sGC *mutants

We analyzed the effects of three *sGC *mutations on the levels of expression of *syt.eGFP *and *n-syb.eGFP*. We used stocks from the *Bloomington Center *that express the hybrid proteins *syt.GFP *or *n-syb.GFP *under the control of *UAS/Gal4 *(*Gal4 *is driven by the specific neuronal promoter *elav*) and located on the X chromosome. The progeny bearing the homozygous *sGC *mutation (associated with recessive markers *ca *and *bv *on the third chromosome) and fluorescence (one copy of the transgene *elav-Gal4-UAS syt.GFP *or *n-syb.GFP *on the first chromosome) were analyzed. Double heterozygous *hsp-sGC*/(;)P [GawB] elav [C155], P [UAS-syt.eGFP], w* and hsp-sGC/(;) P [GawB] elav [C155], P [UAS-n-syb.eGFP], w* were heat-shocked (day 3). Fluorescence in the soluble extract was measured two days later (day 5). Controls were heterozygous flies, *+/"GFP"*. Similarly, we measured the fluorescence intensity in the doubly heterozygous *n-syb.GFP/rut *or *dnc *and *syt.GFP/rut *or *dnc. rut *codes for a cyclase isoform and *dnc *for a phosphodiesterase (both are on the X chromosome), so that the cAMP levels in the two strains are markedly different, although learning and memory behaviour showed identical defects [10,11]. Only females of these strains were considered for analysis. The strains P [GawB] elav [C155], P [UAS-syt.eGFP], w* (*Bloomington Center*, strain number 6923 donator *Kendal Broadie*) and P [GawB]elav [C155], P [UAS-n-syb.eGFP], w*/FM7 (*Bloomington Center*, strain number 6920, donator *Kendal Broadie*) were crossed with *sGC *mutants. Fies (F3, F4) showing *ca *and *bv *along with fluorescence were selected. Strain 6920 is homozygous lethal, so strain *GFP*; s*GC*, homozygous for the mutation, is heterozygous for fluorescence. Strain 6923 is homozygous viable, so in this case we used *GFP;sGC *males in the indicated experiments. One hundred fly heads were ground in a glass Potter and the extract (500 μl) was centrifuged (10000 rpm for 1 h at 4°C). Fluorescence measurements were carried out on the supernatant using a 90 well plate with 100 μl of the extract in each well. The *GFP *domain is attached to the C-terminal of *syt *so the fluorescence is expressed on the cytosolic side of the vesicle membrane (*syt *has its N terminal in the vesicle lumen), while the n-*syb-GFP *construct expresses fluorescence inside the vesicle (*syb *has its C terminal in the vesicle lumen and its N terminal on the cytosolic side). In this latter case, the supernatant of the extract was briefly sonicated before fluorescence was measured.

We used no chemical reagent for protein dosage (such as *Biorad*) of the crude head extract because eye pigmentation interferes. We started with equal number of heads and we verified protein concentrations as follows: a densitometric analysis of a few typical bands was performed after acrylamide gel electrophoresis, colloidal Coomassie blue staining and a 10% acetic acid/25% methanol wash for 24 hours. The concentrations were normalized with a control value (*C-S *fly) as reference. The protein contents of the samples differed by less than 10%.

### Immunoprecipitation analysis of *syt *in *sGC *mutants

One hundred heads were cut off with a razor (flies are immobilized by a brief pulse of CO_2_) and ground in a glass Potter in 500 μl 20 mM phosphate buffer, pH = 7, *plus *1 μg/ml PMSF. This extract was centrifuged 10000 rpm at 4°C for 1 hour and the supernatant was incubated with anti-*GFP *agarose. The pull-down material was then solubilized with 0.5% nonionic detergent (*Tween 20 *1%(*Sigma Aldrich*) in 20 mM phosphate buffer, pH 7, *plus *1 μg/ml PMSF). After brief centrifugation, the supernatant was submitted to immunoprecipitation using anti *syt plus *anti rabbit agarose (I hour incubation with anti *syt*, then with anti rabbit agarose under constant agitation for 1 hour in 20 mM phosphate buffer, pH 7, *plus *1 μg/ml PMSF). The pellet was then washed twice in buffer without detergent and submitted to gel electrophoresis analysis.

### Determination of kinase activity

The kinase activity was assayed as described elsewhere using ^32 ^γP-ATP and histone IIb as substrate [12,13]. Briefly the amount of radioactive phosphate incorporated in histone IIb is measured using a *beta *counter.

### Analysis of *syt*-GFP and *n*-*syb*-GFP in the axonal bundle of the wing margin in *sGC *mutants

We analyzed the fluorescence of the hybrid molecules (*syt-GFP *and *n-syb-GFP*), placed in a *sGC *genetic background as described above (see analysis of *syt *and *n-syb *synthesis), in the wing margin axonal bundle. Wing of flies were examined at day 5. The rescue *hsp-GC *in *GC207ca *background was analyzed as double heterozygous (*hsp-GC plus syt.GFP or syb.GFP*). For these strains, flies were heat-shocked at day 3 and analyzed two days later.

## Results

### Performance in exploration is altered in *sGC *mutants

In a previous paper we reported that searching behaviour involves a strong collective component and flies help each other through various signs to optimize the efficiency of exploration [1]. In other words, we showed that individual flies tested one by one were distributed randomly in a chamber, very different from the distribution obtained with a population of flies. Individual flies were counted in three compartments: immobile on surfaces, flying in the chamber, inside a pierced tube with food. We observed no evolution of the frequencies with which flies were present in these three compartments over a 5-hour time course. Furthermore, these frequencies were to some extent similar in all the strains tested including the *sGC *mutants (see Additional file 2 and the following paragraphs). We then assessed the collective ability of the flies to find a hidden food source in a misleading environmental context. Briefly, two systems compete for the attention of the flies in a chamber. One system consists of a pierced tube; the flies have to pass through the holes to reach a grape juice reward placed inside. The other system is the same except that it includes a layer of glass wool to prevent access. Controls show that the ability of *sGC *mutants to aggregate was unaffected (figure 1). We analyzed the kinetics of distribution and found that most mutants and *C-S *(wild type) flies, along with control *ca *or *bv *flies, stay in the unfavourable tube for only a limited time. When the experiment is carried out with *sGC *mutants, equal numbers of flies remain for longer in both tubes (see figure 2). These mutants are trapped in the "forbidden" system by the odour gradient and it is obviously difficult for them to leave it and find the nearby "good" system. The other strains entering this system leave quickly and colonize the "good" system. In the same context, we found that heat shock-induced expression of *sGC *during metamorphosis (single acute heat shock delivered at the late third instar stage), then during the adult age (single acute heat shock delivered to 3 day old flies), significantly restored the wild type phenotype (see figure 2). The complexity of the tasks performed (directional flight, detection of gradient, finding the holes) by most of the strains tested, including the *sGC *mutants, eliminates major sensory defects in these flies (visual, olfactory, acoustic). Sensory cells are not likely to account for the flaws in searching observed in the *sGC *mutants.

### *sGC *mutants disconnect exploration from detection of odour

**Figure 3:**
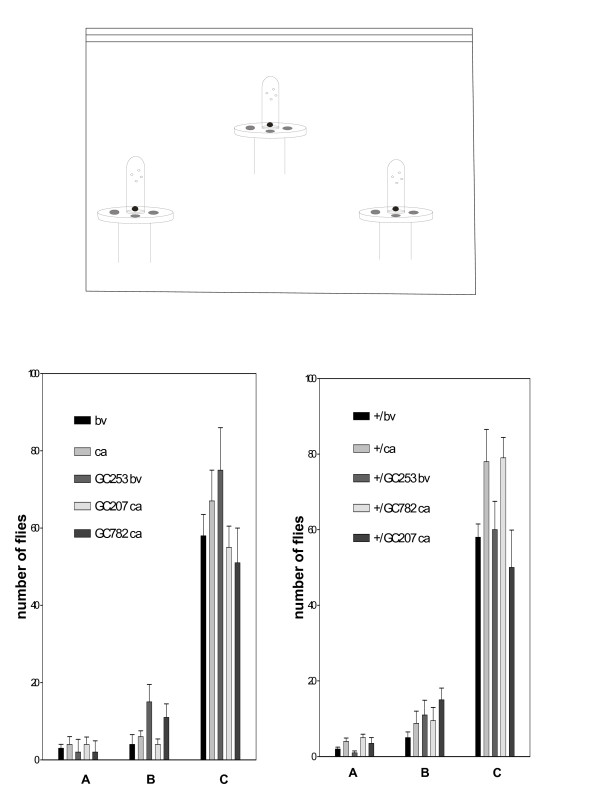
**Exploration/aggregation in odours gradients**. We used a chamber and three systems as detailed in the drawing (*top*). Grape juice (50 μl, 100 μl and 300 μl) was placed inside the three systems. The protocols are described in the Methods section. Briefly, tubes are painted black to eliminate any visual interference with exploration. One set of experiments is carried out in an aerated chamber (odour gradients are maintained), the other in grape juice-saturated atmosphere (see figure 4 and Methods section). One hundred flies were placed in the chamber and the flies inside the 3 tubes were counted 10 hours later. (A) tube with 50 μl, (B) 100 μl and (C) 300 μl. The mutants *GC253bv*, *GC207ca *and *GC782ca *were analyzed along with genetic background *ca *and *bv*. Values are mean ± SEM (n = 10).

**Figure 4:**
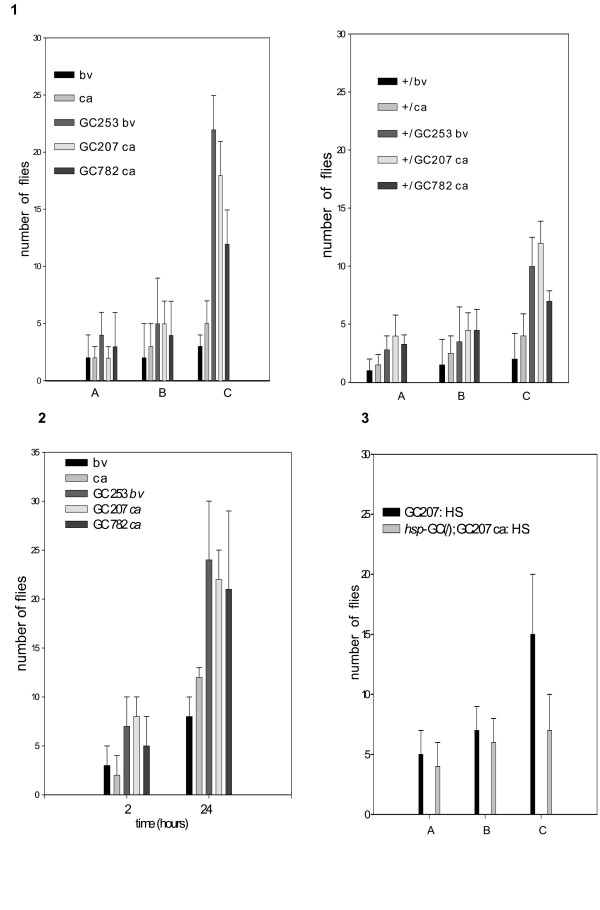
***sGC *mutants disconnect exploration from detection of odour**. (1) Same experiment than in figure 3 except that experiments were carried out in odour-saturated atmosphere. In tube (C), homozygous mutants *versus *their genetic background give: T value 3.87, P = 0.001, degrees of freedom: 18 *(GC253)*; T value 7.6, P = 0.0001, degrees of freedom: 18 (*GC207*); T value 4.36, P = 0.0004, degrees of freedom 18 (*GC782*). The heterozygous mutants show a similar trend (n = 10). Statistics for mutants *versus *genetic background in tube C: T value 4, P = 0.001, degrees of freedom: 16 (*GC253 *and *GC207*); T value 1.98, P = 0.06, degrees of freedom: 16 (*GC782*). (2) The experiment was also performed using a derivative protocol: one single pierced transparent tube was used with 1 ml grape juice in a saturated atmosphere. We observed progression of the accumulation from 2 to 24 hours. Values are mean ± SEM (n = 10). For *GC253bv versus bv *at 24 hours: T value 1.8, P value: 0.085, degrees of freedom: 18 (3) Analysis of the *GC207 *mutant and the rescue *hsp-sGC, GC207 *(protocol as in (1)). The rescue (*hsp-sGC, GC207)*, along with *GC207*, exposed to single acute heat shock at late third instar larva *plus *3 day old adult stage, showed reverse phenotype (similar to control genetic background). Statistics for the mutant *versus *the rescue in tube C: T value 3.42, P = 0.003, degrees of freedom: 18 (n = 10).

*Drosophila *have highly efficient machinery for exploring the environment, and in the course of exploration they simultaneously integrate olfactory, acoustic and visual signals to guide their journey. We used an odour-saturated atmosphere to evaluate how odour gradients guide exploratory behaviour in mutants. We first carried out experiments to analyze aggregation in an atmosphere aerated to preserve the odour gradient emitted from a source of grape juice (figure 3). A parallel protocol was performed in a chamber with a grape juice-saturated atmosphere that eliminated gradients (see figure 4). The final aggregates were counted in each tube. As expected, the most favourable tube (more grape juice) attracted the larger aggregates in the aerated atmosphere (figure 3). When the experiment was carried out with the gradient abolished, we observed that most mutants tested, and the wild type fly (*Cantonese S*.), lost their exploratory behaviour and were unable to find the food sources. In odour-saturated air, the *sGC *mutants were significantly more capable of finding the hidden source without environmental olfactory cues for guidance (figure 4). Remarkably, the behaviour of the *sGC *mutants suggests that their exploratory capacity is not eliminated by the absence of gradient, as we might expect, so it is partially independent of olfaction. The rescue (third instar larvae then 3 day old adults exposed to a single acute heat shock) restores wild type behaviour (figure 4). Our data might be consistent with more aggressive and disorganized exploratory behaviour and might accord with published accounts of the behaviour of *sGC *mutant larvae, showing hyper-locomotion [6]. However, we found that the most favourable spot was systematically more populated with *sGC *mutants in the saturated atmosphere, which suggests that unguided and random visiting of sites does not exclude secondary sub-social aggregation. We therefore conclude that *sGC *mutants disconnect exploration from the detection of odour gradients. We think that the protocol includes its own internal controls for the minimal functionality of the peripheral sensory systems. These data suggest regulation/control of exploration *via *the NO/cGMP pathway.

### *sGC *mutants disconnect taste and aggregation behaviour

**Figure 6:**
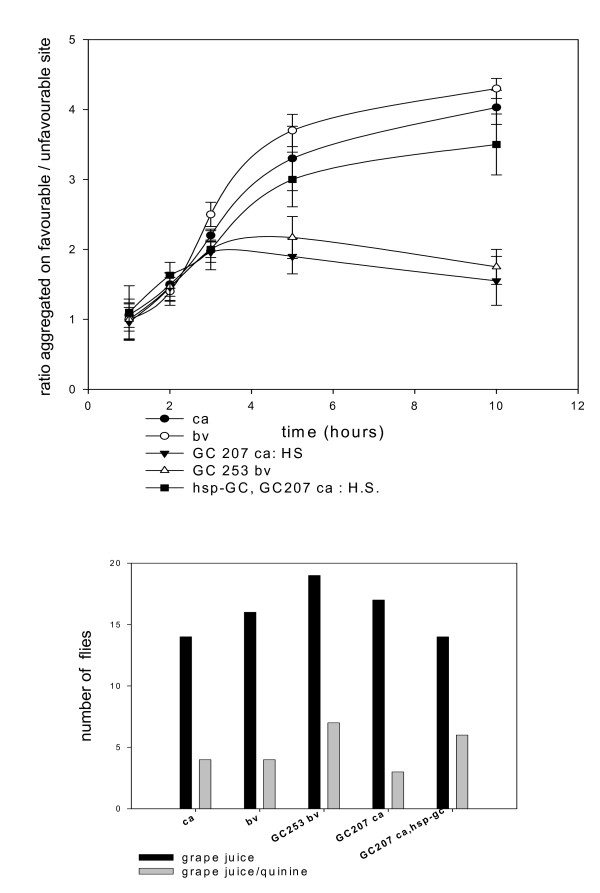
***sGC *mutants disconnect taste and aggregation behaviour**. The time course of aggregation on the two spots was analyzed with 200 flies. We used a chamber and two spots of grape juice. One spot was complemented with denatonium, conferring a bitter taste (see the drawing in figure 5, and Methods section). (*top*) The graph represents the time course of the ratio of numbers of flies on grape juice *versus *denatonium. *GC207ca *and *GC253bv *were tested along with the rescue *hsp-sGC; GC207*. Values are mean ± SEM (n = 10). Statistics for mutants *versus *genetic background at 10 hours: T value 2.32, P = 0.03, degrees of freedom: 18 (*GC253*); T value 2.91, P = 0.01, degrees of freedom: 18 (*GC207*). We observe that rescue (double heat shock: at late third larval stage and 3 day old adult) significantly restores the initial phenotype. (*bottom*) We tested bitterness recognition by the taste sense organs. Thirty flies were analyzed individually after they had been starved for 5 hours (received only water/agar). They were placed in the chamber and had to choose between two alternatives to feed (grape juice spot or grape juice/quinine spot) (see drawing in figure 5). Flies tended to fly away after proboscis extension when they detected quinine. Arbitrarily, we counted flies feeding for more than 10 seconds on a spot.

*Drosophila *aggregate on food spots; the evolutionary advantage of this is that females in aggregates can screen more males for mating. Furthermore, females have to assess the quality of food resources in order to lay eggs. Interestingly, the choice of aggregation spot is a reliable collective evaluation of the food resources necessary to sustain larval development [14,15]. Therefore, we reasoned that aggregation on a misleading and unfavourable spot in competition with a favourable spot could highlight a collective "mistake" in performing an aggregation task. We designed an experimental protocol as follows: two spots of identical grape juice food were placed in a chamber and constituted alternative choices for the attention of the flies. The only difference was that one was complemented with denatonium, which confers a bitter taste. The control showed that the total aggregated *versus *free in the chamber was of the same order in the different strains tested (figure 5). We checked then that the bitter compounds are repulsive, and observation showed that recognition of bitter molecules is little affected by the mutations tested, including *sGC *(figure 6). Using this protocol, we found that the *sGC *mutants aggregated significantly on the bitter spot during the time course of the experiment, whereas the controls flocked on the favourable spot (see figure 6). The rescue (double heat shock delivered to third instar then 3 day old adult) significantly corrected the phenotype (figure 6). The control showed that the total aggregated *versus *free in the chamber was of the same order in the different strains tested, which again supports the inference that *sGC *mutations induced flaws in coincidental neuronal signalling. This suggests that *sGC *mutants partly disconnect taste from aggregation.

### *sGC *mutants show sub-social aggregation defects

**Figure 7:**
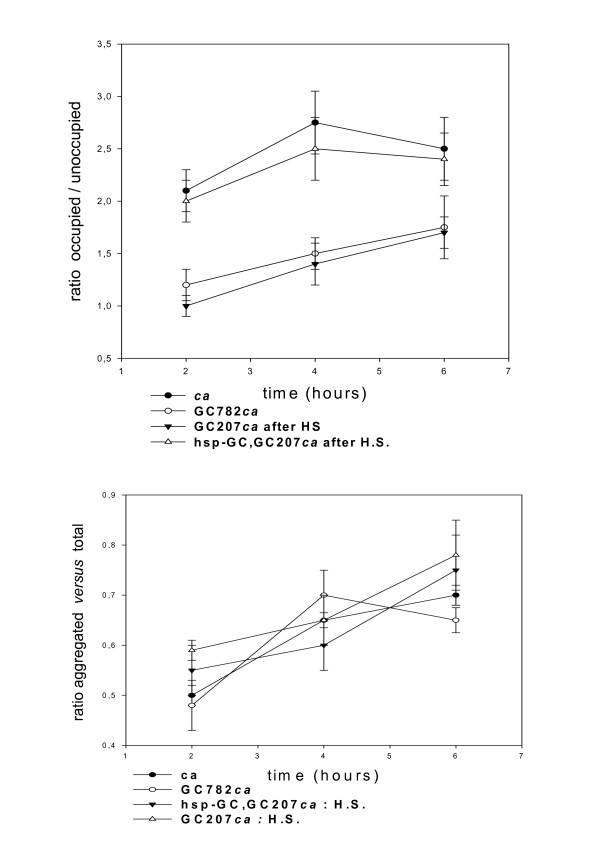
***GC *mutants show sub-social aggregation defects**. (*top*) Social aggregation of two mutants *GC207 *and *GC782*. Pre-aggregated *w1 *flies (50 flies ± 10) were used to "lure" mutants. Mutants (20 flies, 5 days old, red eyed, females to avoid pheromone interference) were released in the chamber without disturbing the aggregated spot (*w1 *aggregates stable, probably because of altered vision). Simultaneously, a second fresh spot was placed inside. Here, the graph represents the time course of the ratio of aggregated flies (*GC 207 *or *GC 782*) on the pre-aggregated spot *versus *the fresh one. Values are mean ± SEM (n = 10). The *GC207 *mutant against control *ca *gave T value: 3.38, P = 0.003 and degrees of freedom: 18. Heat shock rescue was carried out as in figure 1. (*bottom*) The control of flies aggregated *versus *the total in the chamber is represented for the strains tested above. We saw no significant differences between the strains. The apparatus was the same than in the figure 5 and 6.

We also tested our mutants using a variant experimental design. We used the same two spots of grape juice food in a chamber except that one was already aggregated with *w1 *flies. In other words, we tested the behaviour of our mutants in the presence of pre-aggregated *w1 *flies. This strain is recognizable by the white eye colour, and we reported in a previous article that their altered vision triggers long-lasting and stable aggregation [1]. *sGC *mutants (*GC207 *and *GC782*) showed no significant preference between the aggregated spot and a virgin fresh alternative. In this test, other stocks tested and the control fly *ca *showed a tendency to flock on the already-aggregated spot (see figure 7). The controls showed that the ratio of total flies on food spots to flies free in the chamber was of the same order in all the strains tested (figure 7), and this again allows us to eliminate major peripheral sensory defects (olfactory or visual). This experiment shows that *sGC *mutants have an altered sub-social component for guiding exploration (see ref. 1 for arguments in favour of cooperative searching in *Drosophila*).

### Analysis of neural circuitry of coincident signalling involved in exploration/aggregation behaviour

We then tried to identify the cells involved in these behaviours. We failed to uncover the relevant circuitry because *sGC *is ubiquitous in the brain. Immunohistology using an anti-*sGC *antibody showed no specific labelling in any anatomical part of the brain (data not shown). *sGC *mutants have no structural brain defect as far we could observe by microscopy (data not shown). The cellular mass was identical to wild type, as measured by quantification of a neuronal marker recognized by anti-HRP antibody (data not shown). To approach the molecular mechanisms involved in these behavioural defects, we performed biochemical experiments with adult brains of *sGC *mutants and the rescue (induction of *hsp*-*sGC *at adult age in mutant background). We pursued this work although the neurons involved in the coincidence systems are probably "diluted" in the CNS. We analyzed the effects of three *sGC *mutations on the levels of expression of *syt.eGFP *and *n-syb.eGFP*. We found that *sGC *mutations slightly increase the levels of fluorescence of the two hybrid molecules, although the global effect is modest. Moreover, the rescue (induction of *hsp*-*sGC *at adult age in mutant background) diminishes significantly the level of fluorescence of the two hybrid molecules (see Additional file 3). We didn't see differences in *sGC *mutants and control regarding the amount of the three isoforms of *syt *molecules (see Additional file 4). However two *syt *isoforms were down regulated after overexpression of *sGC *in the rescue after heat shock. This argues in favour of a role for *sGC *to regulate the synthesis and/or transport of synaptic vesicle components along the axons after wiring.

**Figure 8:**
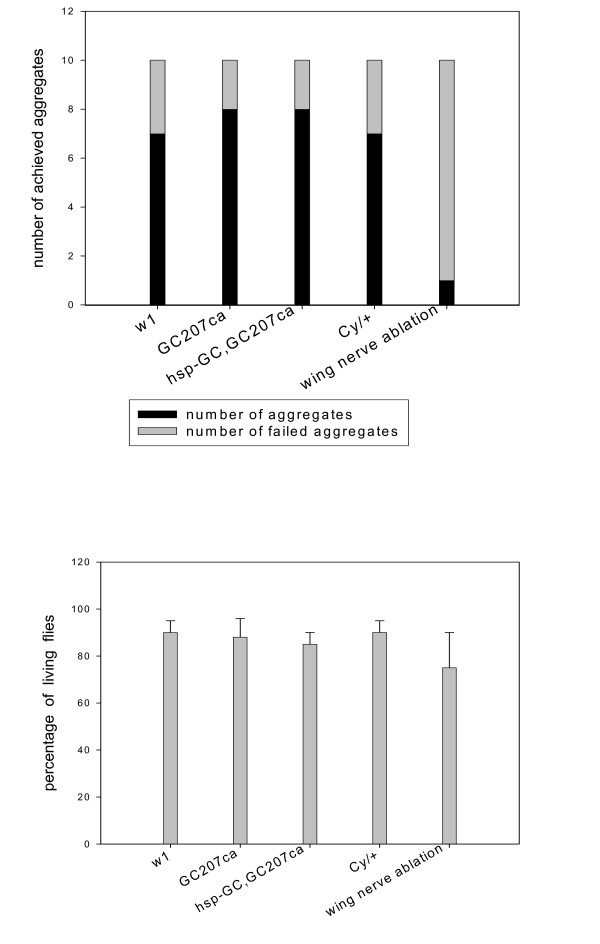
**Analysis of a simple coincidence system in *sGC *mutants: the axonal bundle in the wing margin **. (*top*) The nerve was sectioned with a razor in the proximal part of the left wing of *C-S *under C0_2 _anaesthesia. The flies (100) were tested one day after the surgery using a derived aggregation protocol (a chamber *plus *a food spot placed on the bottom). As controls, we report a comparative assay with the *w1 *mutant (altered vision), *Cy *(impaired flight) along with *sGC *mutant (GC 207) and its rescue (after heat shock). Ten experiments were carried out. The graphs represent the number of experiments showing aggregation above 50% of the total after 5 hours. (*bottom*) A comparative test of lethality with 50 flies of the indicated strains (8 days old flies) and the nerve sectioned strain carried out 5 days after the surgery.

In view of this complexity, we decided instead to focus on a simple coincidence system, the sensory organs of the wing margin, which has the advantage that two types of sensory neurons are entangled in the same axonal bundle: mechanoreceptors and chemoreceptors [2-5]. This simple system is highly accessible and relevant to flight guidance. Unilateral lesion of the wing margin nerve of *C-S *abolishes the aggregation phenomenon and the collective behaviour of these flies resembles that of individual flies. We deduced that the integration of signals generated by sensory cells in the wing during flight contributes to the fly's synchronized "decisions" (see figure 8).

**Figure 9:**
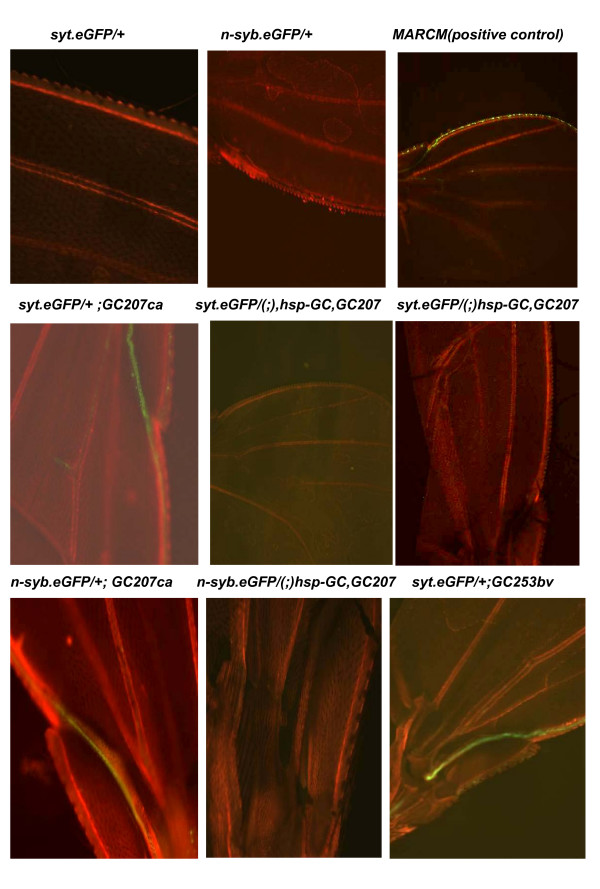
**Synthesis/transport of synaptic vesicle in wing nerve of *sGC *mutants**. The strains P [GawB] elav [C155], P [UAS-syt.eGFP], w* and P [GawB] elav [C155], P [UAS-n-syb.eGFP], w*/FM7 were crossed with *sGC *mutants (see materials and methods section). Flies bearing one copy of hybrid GFP (first chromosome) and homozygous for *sGC *mutations were analyzed for the fluorescence in the wing. The rescue *hsp-GC *in *GC207ca *background was analyzed as double heterozygous (*hsp-GC plus syt.GFP or syb.GFP*). Flies were heat-shocked at day 3 and analyzed two days later. Positive control: MARCM system (chromosome recombination based on flipase under heat-shock promotor induces axonal *GFP *marker -*Bloomington Center stock 5144 and 5134*).

Moreover, we reasoned that specific mechanism(s) and molecular machinery involving *sGC *might influence the mutual interaction of the two types of neuron in order to achieve efficient exploration. We therefore analyzed the fluorescence of hybrid molecules expressed in synaptic vesicles in the wing margin axonal bundle (*syt-GFP *and *n-syb-GFP*) placed in a *sGC *genetic background. The results, reported in figure 9, show evidence for a role of the NO/cGMP pathway in this neuronal system. We observed that *syt-GFP *was up-regulated in these types of neurons only in *sGC *mutants whereas the overall fluorescence in the brain was little changed as reported above.

## Discussion

Many reports have described the genetic complexity underpinning foraging behaviour, especially in *Drosophila *[16-18], and an abundant literature reports the successful application of genetic tools in deconstructing complex behaviours in model animals [19-21]. Complex behaviours involve brain integration of multiple environmental signs in order to guide decisions. Flies might disregard some signs and overreact to others depending on the context. In this sense, the paradigm of molecular sensors of coincidence is advantageous in linking molecules to complex behaviour [22]. We carried out this work because the well-documented foraging genes in *Drosophila *and honeybee code for cGMP-dependent kinases, and pleiotropic effects are induced downstream of their activation [12,13,23]. By better integrating the multiple environmental signs sensed by the neurosensory systems (visual, acoustic, olfactory, tactile), explorers variants (honeybee, *drosophila*) should be more equipped to face the adversity and hostility of the environment. In this report, we used tests designed to evaluate whether certain mutations in key molecules can specifically affect the integration of multiple neuronal signals that contribute to enabling flies to perform stereotyped tasks in exploration. We considered that flies naturally (innately) perform tasks involving skills and strategies for finding food and sexual partners, otherwise they would wander in the environment. The stereotyped tasks forming the basis of our tests constitute cycles of exploration/aggregation. Although highly complex gene networks and pleiotropy account for these complex behaviours, the phenomenon of coincidence highlighted in our tests seems to relate to a more restricted subset of genes. We screened more than twenty mutants of genes involved in neuronal signalling (Cam kinase, PKC, PKG mutants, mutants of synaptic machinery; see the list in Additional file 1) and found that mutants of *sGC *(along with *dnc *mutants) present a marked behavioural phenotype. Abundant literature documents the role of *sGC *in neurobiology. Nitric oxide (NO) diffuses as short-lived messenger through the plasma membrane and serves, among many other functions, as an activator of the soluble guanylyl cyclase (sGC) [24,27]. Authors have reported that NO could affect the steering decisions of growth cone during neuronal pathfinding [28]. Furthermore, authors have described that presynaptic elements are assembled into 'prototerminals' before being transported down the axons and these structures stop their migration when the axons contact a dentrite [29]. Interestingly direct application of a NO donor to neurons resulted in inhibition of axonal transport of synaptophysin and synaptotagmin I tagged with EGFP in neuron culture [30]. Authors reported also that *sGC *is asymmetrically localized to the developing apical dendrites in mammal neurons and is required for the chemoattractive effect of some molecules [31]. Authors have reported also a role of NO as "stop and go" signal for neuronal processes [32,33], a role in communication between sensory afferent neurons [34], in long term depression [35,36] and in retrograde signal to regulate the synaptic vesicle recycling [37-39]. All together these elements argue for the presence of fragile and/or misguided synapses in our *sGC *mutants. The wing nerve analysis allowed us to evaluate that *sGC *mutations result in a continued synthesis and/or transport of synaptic vesicle components, which should be stopped by proper axon/dentrite wiring. The *sGC *mutants are viable. *sGC *is therefore not essential gene for major developmental steps. However, we were able to see obvious effect of this gene on the neuronal coincidence signalling based on behavioural tests. Our behavioural data supports the conclusions of a deficiency of integration of signals emanating from neurosensorial systems.

## Conclusion

As distinct from learning and memory, our paradigm mostly concerns synchronized fly "decision making" resulting from the simultaneous processing of environmental signs. We might suggest by deduction that *sGC *mutants appear to unravel the function of this gene in neurons involved in integrating and synchronizating cellular signals. On the other hand, the adult wing of *Drosophila*, equipped with sensory bristles which are mechanoreceptors and chemoreceptors, is highly accessible. This constitutes a simple coincidence system. Although biochemical analysis revealed no detectable major defects in the brains of *sGC *mutants, the wing nerve shows altered regulation of molecular components, which might account for the exploratory disorders.

## Abbreviations

*sGC: *soluble guanylate cyclase. *hsp: *heat shock promoter. *rut: *rutabaga. *dnc:*dunce. *syt.eGFP: *synaptotagmin/green fluorescent protein fusion. *n-syb.eGFP :*synaptobrevin/green fluorescent protein fusion. *NO: *nitric oxide. *NOS: *Nitric oxide synthase.

## Authors' contributions

ST and AR did the behavioural experimental work. AG did biochemistry work (specially phosphorylation and immnunoprecipitation). GE did the fluorescence analysis AR did the experimental designs of experiments and ST contributed through discussion and data analysis.

## Supplementary Material

Additional file 1 List of mutants tested in this study. These strains were tested using the protocol described in figure 1 and only the *sGC *mutants showing strong phenotype were further investigated. Click here for file

Additional file 2 Analysis of distribution of individual flies (*sGC *mutants and rescue) in a chamber. fly were placed one by one in a chamber and analyzed for their location over a period of time of 5 hours. Click here for file

Additional file 3 *sGC *slightly modulates the synthesis of synaptic vesicle proteins. The constructs homozygous for the *sGC *mutation (third chromosome) and bearing one copy of the syt-GFP or syb-GFP construct (first chromosome) were analyzed for relative fluorescence intensity. Click here for file

Additional file 4 analysis of *syt *isoforms in *sGC *mutants. Content of *syt *isoforms in the immuno-isolated synaptic vesicles of *sGC *mutants was analyzed in acrylamide gel electrophoresis. Click here for file
