# Supplementary material for: Exploratory behaviour in NO-dependent cyclase mutants of Drosophila shows defects in coincident neuronal signalling
Source: BMC Neurosci. 2007 Aug 6;8:65. doi: 10.1186/1471-2202-8-65 (PMC1963332; doi:10.1186/1471-2202-8-65)
Supplement: Additional file 1 — List of mutants tested in this study. These strains were tested using the protocol described in figure 1 and only the sGC mutants showing strong phenotype were further investigated. [file 1471-2202-8-65-S1.pdf]

List of strains tested in this study

Ala2: speudosubstrate inhibitor of Cam kinase II under heat shock promotor (*ref a*)

RQED: transgene Cam kinase II under heat shock promotor (*ref a*)

For: natural mutant showing Rover behaviour and high expression of long transcripts of PKG gene DG2 (*ref b*)

164: sitter behaviour mutant, shows low level of PKG activity (*ref b*)

fors2: other sitter behaviour mutant (*ref b*)

Y2-2: transgene *for* (PKG activity) under heat shock promotor in the *164* background (*ref b*)

6923: P[gaw B] elav [C155], P[UAS-syt.eGFP]1, w* syt.eGFP is an hybrid molecule synaptotagmin/GFP (*Bloomington center*)

6920: P[gaw B] elav [C155], P[UAS-n-syb.eGFP]&,w*/FM7 syb.GFP is an hybrid molecule synaptobrevin/GFP (*Bloomington center*)

MARCM system: P[gaw] elav [C155]w* P[neoFRT] 19 A,
And P[neoFRT] 19A, P[tub-gal80]LL1, P[hsFLP]1, w*; P[UAS-mCD8::GFP]LL5
8 lines corresponding to heat shock procedure at **8** different stages of larvae and pupae generated different patterns of fluorescence in neurons at adulthood. (*Bloomington center*) (ref c)
5130: UAS mCD8::GFP on X was crossed with a *Gal4 elav* (*Bloomington center*)

5136: UAS mCD8::GFP on chromosome 3 was crossed with a *Gal4 elav* (*Bloomington center*)
tur: PKC activity defect (*Bloomington center*)

dnc: phosphodiesterase defect, high level of cyclic AMP (*Bloomington center*)

rut: cyclase defect, low level of cyclic AMP (*Bloomington center*)

am: amnesiac, memory defect (*Bloomington center*)

am [EP]: EP line with UAS inserted into the gene allowing over-expression of *am* by crossing with *Gal4 elav* strain (*Bloomington center*)

rut [EP]: EP line with UAS inserted into the gene allowing over-expression of *rut* by crossing with *Gal4 elav* strain (*Bloomington center*)

These lines were tested with the protocols described in figure 1. After five experiments showing little effect compared to control (*C-S*) we decided to stop investigating these mutants in the exploratory paradigm.   a) *J. Cell. Biochem.*1996 Vol **62** 484-494
b) *Science* 1997 Vol **277** 834-36   c**)** *Neuron*   1999 Vol **22:** 541-61
