# Supplementary material for: Exploratory behaviour in NO-dependent cyclase mutants of Drosophila shows defects in coincident neuronal signalling
Source: BMC Neurosci. 2007 Aug 6;8:65. doi: 10.1186/1471-2202-8-65 (PMC1963332; doi:10.1186/1471-2202-8-65)
Supplement: Additional file 2 — Analysis of distribution of individual flies (sGC mutants and rescue) in a chamber. fly were placed one by one in a chamber and analyzed for their location over a period of time of 5 hours. [file 1471-2202-8-65-S2.pdf]

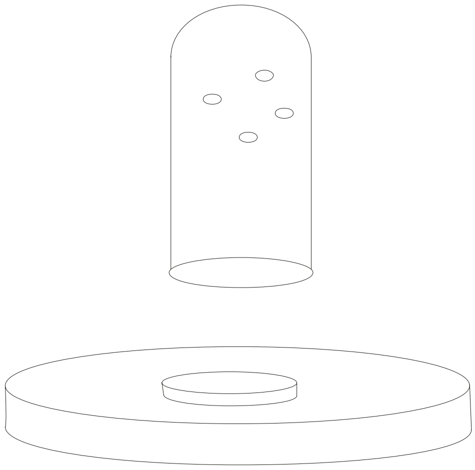

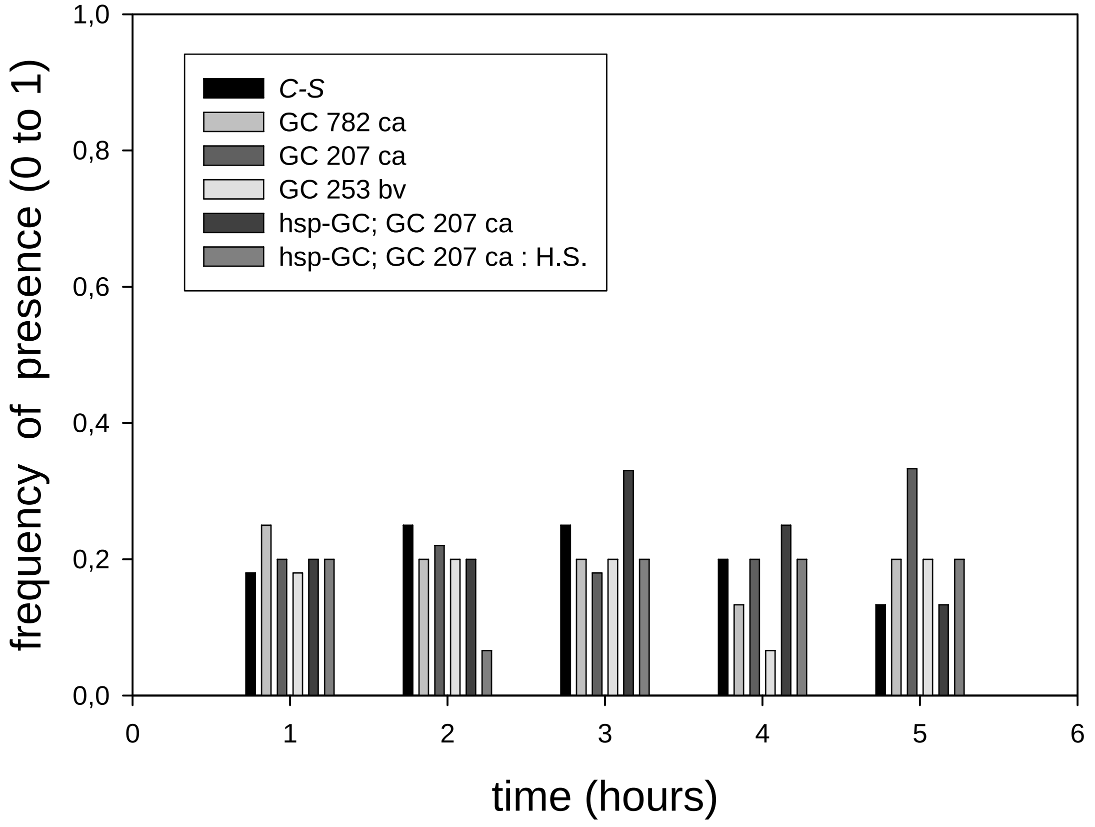

1,0
0,8
0,6
0,4
0,2
0,0
frequency of presence (0 to 1)
C-S
GC 782 ca
GC 207 ca
GC 253 bv
hsp-GC; GC 207 ca
hsp-GC; GC 207 ca : H.S.
0
1
2
3
4
5
6
time (hours)

Analysis of distribution of individual flies (*sGC* mutants and rescue) in chamber

Five day old flies were tested individually in a chamber (see methods). Flies (20 for each strain) were placed one by one inside the chamber (see drawing in figure 1, excepted a unique pierced tube represented at the top was used) and their locations were analyzed each hour. The presence of flies feeding inside the tube was counted. Results are shown as frequencies on a scale 0 to 1 based on the random presence of 20 flies tested individually. We observed no significant trend between 1 and 5 hours for each strain. Moreover the average of the 5 times for each strain gave a score similar between them and no significant difference using the *Student t* test.

Mean of the scores for the 5 times +/- standard error. Statistics were done using *Student t* test against the control *CS* for each strain.
CS: 0.215+/- 0.015
GC 782 ca: 0.205+/-0.012 (T value 0.5, P value 0.61, D.F. 8)
GC 207 ca: 0.23+/-0.02 (T value 0.6, P value 0.56, D.F. 8)
GC 253 bv: 0.18+/-0.02 (T value 1.4, P value 0.19, D.F. 8)
hsp-GC, GC207 ca: 0.211+/-0.032 (T value 1.37, P value 0.89, D.F. 8)
hsp-GC, GC 207: H.S: 0.18+/-0.02 (T value 1.4, P value 0.19, D.F. 8)
