# Supplementary material for: Exploratory behaviour in NO-dependent cyclase mutants of Drosophila shows defects in coincident neuronal signalling
Source: BMC Neurosci. 2007 Aug 6;8:65. doi: 10.1186/1471-2202-8-65 (PMC1963332; doi:10.1186/1471-2202-8-65)
Supplement: Additional file 3 — sGC slightly modulates the synthesis of synaptic vesicle proteins. The constructs homozygous for the sGC mutation (third chromosome) and bearing one copy of the syt-GFP or syb-GFP construct (first chromosome) were analyzed for relative fluorescence intensity. [file 1471-2202-8-65-S3.pdf]

A

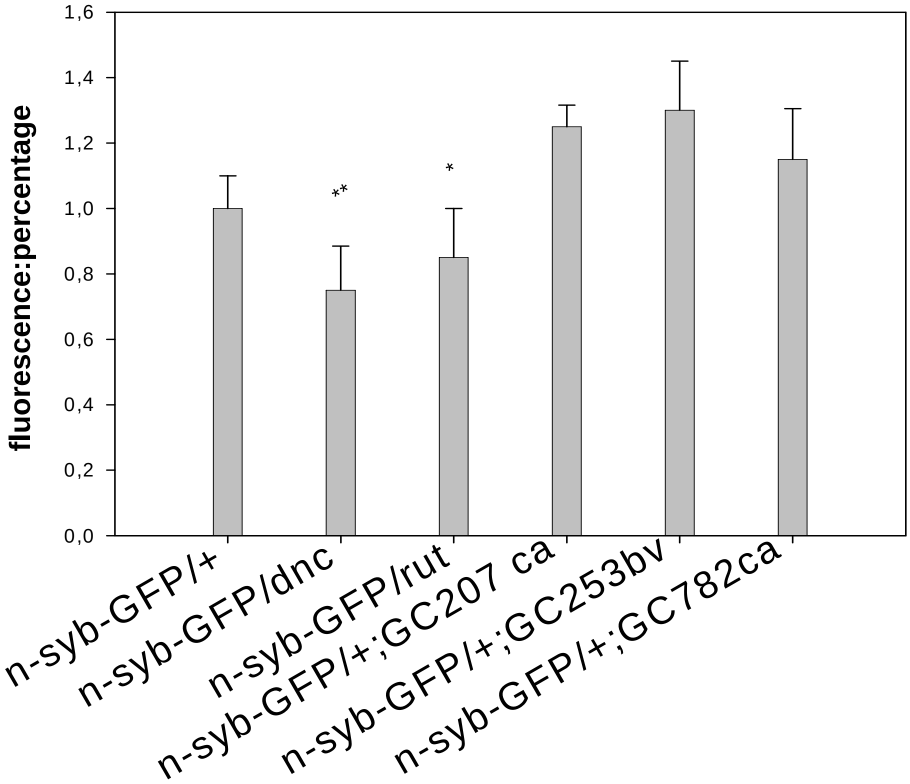
fluorescence:percentage
1,6
1,4
1,2
1,0
0,8
0,6
0,4
0,2
0,0
**
*
n-syb-GFP/+
n-syb-GFP/dnc
n-syb-GFP/rut
n-syb-GFP/+;GC207 ca
n-syb-GFP/+;GC253bv
n-syb-GFP/+;GC782ca

B

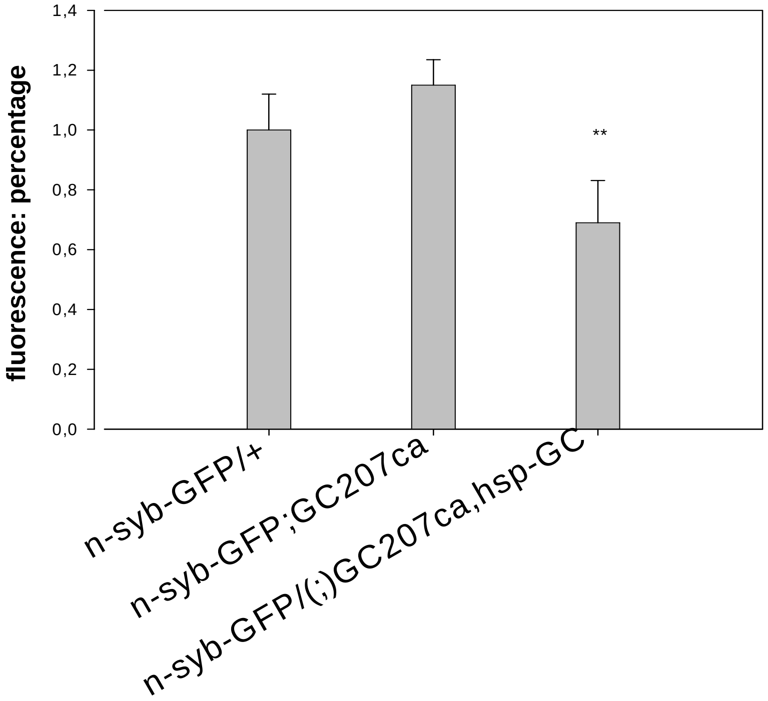
fluorescence: percentage
1,4
1,2
1,0
0,8
0,6
0,4
0,2
0,0
**
n-syb-GFP/+
n-syb-GFP;GC207ca
n-syb-GFP/(;)GC207ca,hsp-GC

C

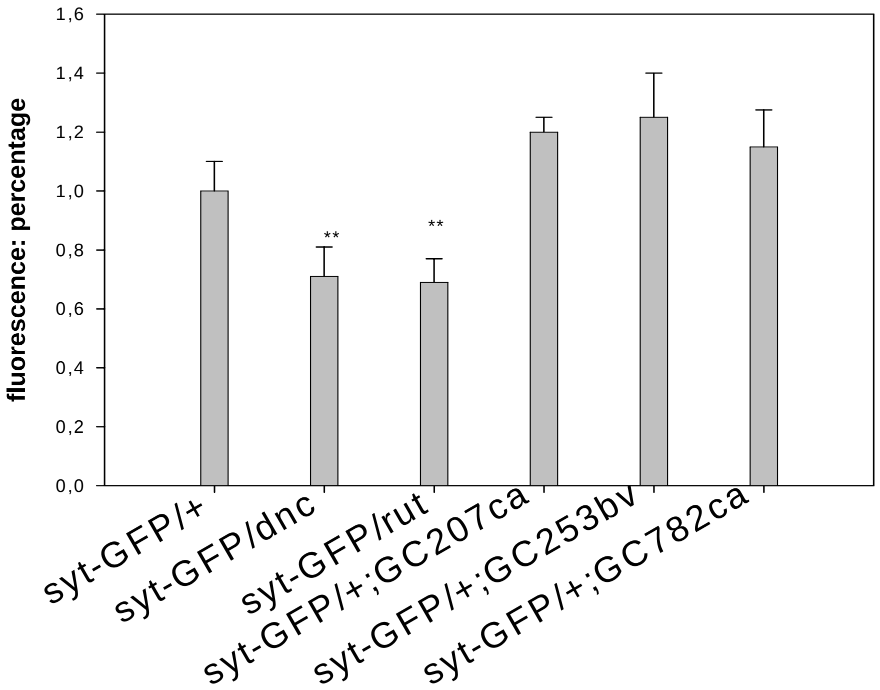

1,6
1,4
1,2
1,0
0,8
0,6
0,4
0,2
0,0
fluorescence: percentage
**
**
syt-GFP/+
syt-GFP/dnc
syt-GFP/rut
syt-GFP/+;GC207ca
syt-GFP/+;GC253bv
syt-GFP/+;GC782ca

D

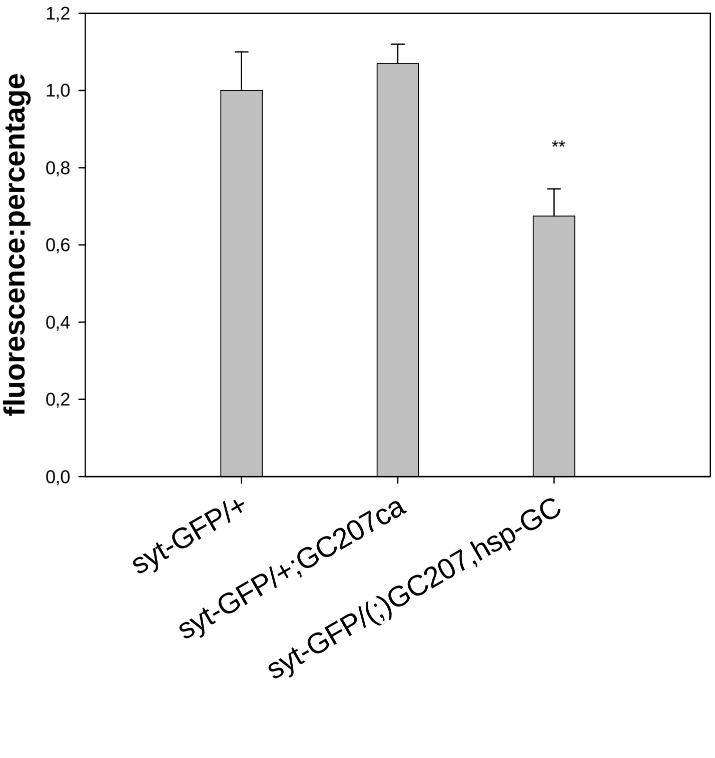

1,2
1,0
0,8
0,6
0,4
0,2
0,0
fluorescence:percentage
**
syt-GFP/+
syt-GFP/+;GC207ca
syt-GFP/(;)GC207,hsp-GC

***sGC* slightly modulates the synthesis of synaptic vesicle proteins**

A,C: *P[GawB] elav[C155], P[UAS-syt.eGFP],w** (*Bloomington Center*, strain number 6923) and *P[GawB]elav[C155], P[UAS-n-syb.eGFP], w*/FM7* (*Bloomington Center*, strain number 6920) were crossed with mutants *GC253bv*, *GC782ca* or *GC708ca.* The constructs homozygous for the *sGC* mutation (third chromosome) and bearing one copy of the GFP construct (first chromosome) were analyzed for relative fluorescence intensity.
Double heterozygous *rut* or *dnc*/ *P[GawB] elav[C155], P[UAS-syt.eGFP],w** and *rut* or *dnc/ P[GawB] elav[C155], P[UAS-n-syb.eGFP], w** were analyzed for comparison (see Methods section for more details).

B,D: Double heterozygous *hsp-sGC*/(;) *P[GawB] elav[C155], P[UAS-syt.eGFP],w* and hsp-sGC/(;) P[GawB] elav[C155], P[UAS-n-syb.eGFP], w** were heat-shocked (day 3) and analyzed (day 5) against the *syt* or *n-syb-GFP* in the homozygous mutation . Controls were heterozygous flies, *+/ "GFP".*

Values are mean ± SEM. Statistics are determined against the control. (**) means $P<0.01$. T value 2.44, $P = 0.025$, degrees of freedom: 18 (*n-syb/dnc*, n=10); T value 2.5, $P = 0.02$, degrees of freedom: 18 (*syt/dnc* and *syt*/*rut*, n=10); T value 3.32, $P = 0.01$, degrees of freedom: 8 (*n-syb/hsp- sGC*, n=5); T value 2.45, $P = 0.039$, degrees of freedom: 8 (*syt/hsp-sGC,* n=5).
