# Supplementary material for: Exploratory behaviour in NO-dependent cyclase mutants of Drosophila shows defects in coincident neuronal signalling
Source: BMC Neurosci. 2007 Aug 6;8:65. doi: 10.1186/1471-2202-8-65 (PMC1963332; doi:10.1186/1471-2202-8-65)
Supplement: Additional file 4 — analysis of syt isoforms in sGC mutants. Content of syt isoforms in the immuno-isolated synaptic vesicles of sGC mutants was analyzed in acrylamide gel electrophoresis. [file 1471-2202-8-65-S4.pdf]

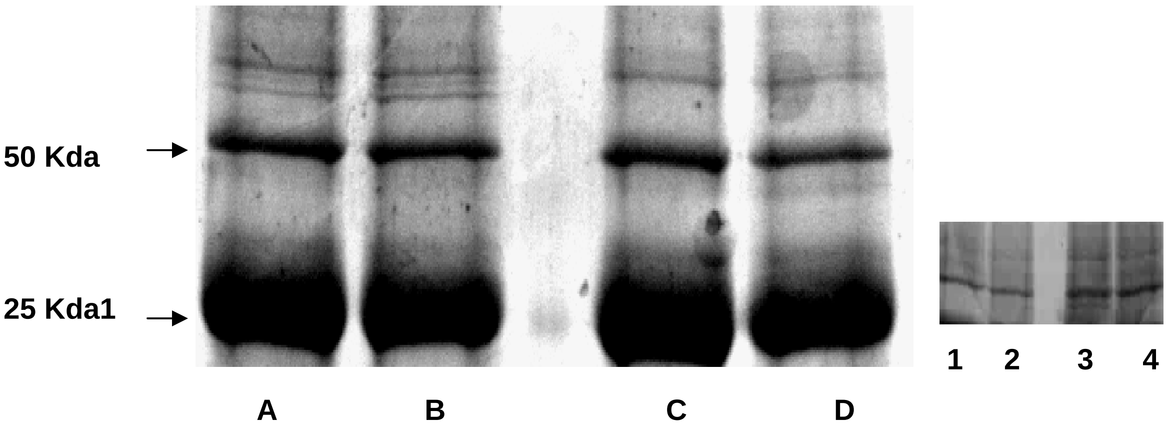

50 Kda
25 Kda1
A
B
C
D
1
2
3
4

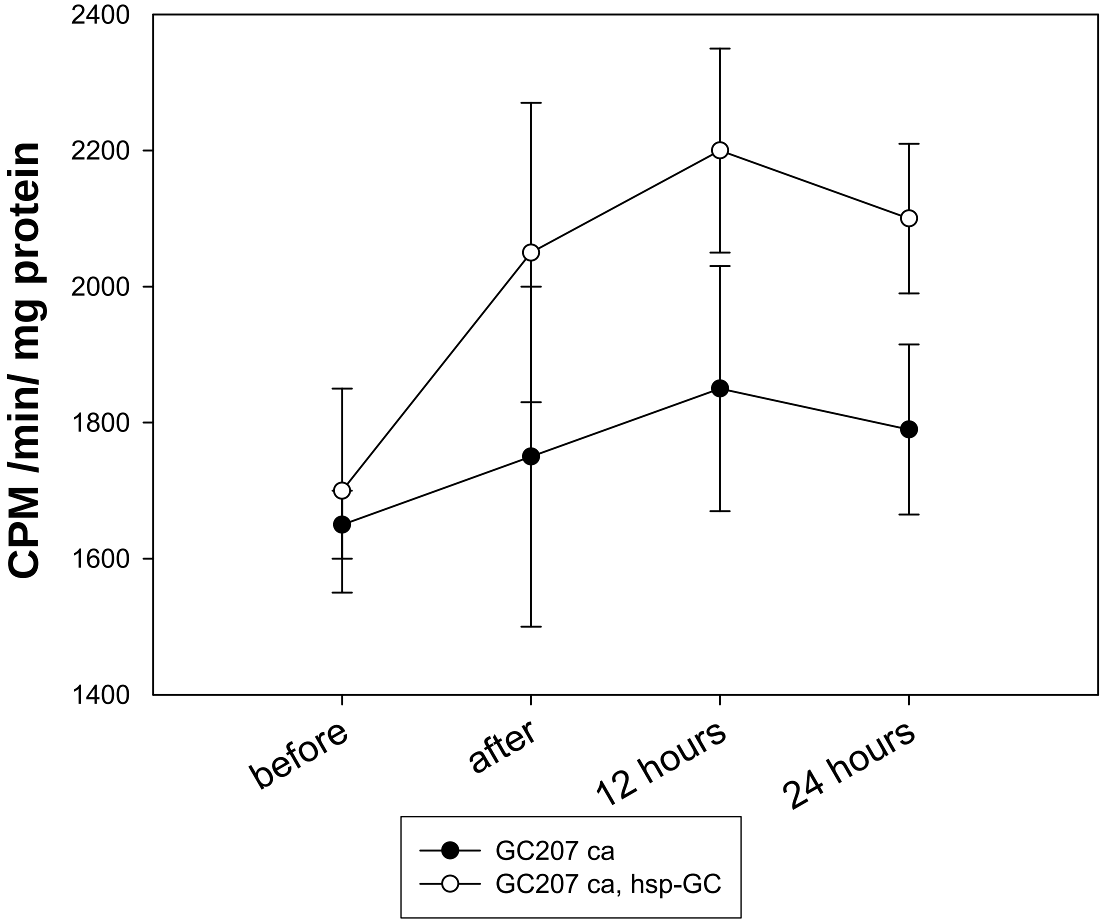

CPM /min/ mg protein
2400
2200
2000
1800
1600
1400
before
after
12 hours
24 hours
GC207 ca
GC207 ca, hsp-GC

(*top*) **Content of *syt* isoforms in the immuno-isolated synaptic vesicles of *sGC* mutants**
Vesicles were immunoisolated with anti-GFP agarose (buffer without detergent). The material was then solubilized and *syt* molecules were immunoprecipitated before gel acrylamide analysis (10%). Lanes A and B: we see 3 bands with *P[GawB] elav[C155], P[UAS-syt.eGFP], w*; GC207ca* or + (homozygous for *GC207ca* or +) 1 day after heat shock. Lanes C and D: one band is observed with *P[GawB] elav[C155], P[UAS-syt.eGFP], w*/(;)hsp*-sGC (double heterozygous) 1 day and 3 days after heat shock.
(right): Control immunoprecipitation with anti *syt* in the crude solubilized extract.
Lanes 1 and 2, respectively: *GC207ca* with and without heat shock,
lanes 3 and 4, respectively: *hsp-sGC;GC207ca* with and without heat shock.
No differences were observed between the *sGC* mutant and the rescue with or without heat shock (only one major band appeared).

(*bottom*) **cGMP dependent kinase activity**
A cGMP-dependent kinase was measured in mutant *GC207ca* and its *hsp-GC* rescue (before and after heat shock). The kinase activity was assayed with head extracts of the mutant and its rescue as described elsewhere using ^{32} γP-ATP and histone IIb as substrate [ref, below]. The activity is reported as the amount of radioactivity of phosphate group incorporated in histone. Control without heat shocked is reported as “before”. Flies were heat shocked for 30 minutes at 37°C then the dosage in the head extract was performed with animals 1 hour, 12 hours or 24 hours after heat shock. Data represent mean ± SEM (n=3) (*bottom*).

Ref: K.A. Osborne, A. Robichon, E. Burgess, S. Butland, R.A. Shaw, A. Coulthard, H.S. Pereira, R.J. Greenspan, M.B. Sokolowski. **Natural behaviour polymorphism due to a cGMP dependent protein kinase of drosophila.** *Science* 1997 **277:** 834-36
